# Machine learning using entropy–based texture features from MRI to differentiate histological subtypes of non–small cell lung cancer identified as metabolically active on PET/MRI

**DOI:** 10.1371/journal.pone.0338373

**Published:** 2026-01-21

**Authors:** Marta Borowska, Małgorzata Mojsak, Ewelina Bębas, Jolanta Pauk, Marcin Hładuński, Małgorzata Domino

**Affiliations:** 1 Bialystok University of Technology, Faculty of Mechanical Engineering, Institute of Biomedical Engineering, Białystok, Poland; 2 Medical University of Białystok, Independent Laboratory of Molecular Imaging, Białystok, Poland; 3 Warsaw University of Life Sciences, Department of Large Animal Diseases and Clinic, Institute of Veterinary Medicine, Warsaw, Poland; The University of Texas, MD Anderson Cancer Center, UNITED STATES OF AMERICA

## Abstract

Texture analysis is a foundational approach in imaging studies and demonstrates excellent diagnostic performance, with radiomic analysis being the most widely used method. New approaches to texture analysis continue to be developed. However, magnetic resonance imaging (MRI)–based radiomics studies for identifying histological subtypes of lung cancer remain scarce. This study aimed to improve the efficiency of the computer–aided non–invasive diagnosis of non–small cell lung cancer (NSCLC) by supplementing the statistical approaches to MRI image texture analysis with entropy–based methods. The study included 31 patients with NSCLC, categorized into two histological groups containing 12 patients (75 images) with adenocarcinoma (ADC) and 19 patients (79 images) with squamous cell carcinoma (SCC). A total of 154 MRI images were annotated using 154 regions of interest (ROIs). ROIs were extracted, filtered using normalize and median filtrations, and analyzed using standard statistical approaches and novel entropy–based methods. Texture features were selected using Select From Model (SFM) protocol and the classified using k–Nearest Neighbors (kNN), Support Vector Machines (SVM), and Logistic Regression (LR), separately. After 5–fold stratified cross–validation, the LR algorithm achieved the highest classification performance (0.75 accuracy and 0.78 presision) on the combined statistical and entropy–based texture features extracted from MRI images after median filtration. The proposed protocol presented higher efficiency than protocols that worked only on the statistical texture features on unfiltered or normalize filtered MRI images; therefore, it may be suggested for further research on the computer–aided diagnosis of NSCLC histological subtypes.

## 1 Introduction

Non–small cell lung cancer (NSCLC) is the second most common cancer globally, accounting for 85% of lung cancer cases and approximately 1.4 million deaths annually [1]. Among diagnoses lung cancers, there are four major histological subtypes: adenocarcinoma (ADC, approximately 40%, the most common subtype in passive smokers), squamous cell carcinoma (SCC, approximately 30%, the most common subtype in active smokers), small cell carcinoma (SC, approximately 15%), and large cell carcinoma (LCC, approximately 10%) [2]. Given ADC and SCC are the primary histological subtypes of NSCLC, their recognition and differentiation are an important clinical challenge that prolongs the patients’ lifespan [3]. Prompt diagnosis of the specific NSCLC histological subtypes, considering their biological characteristics, is crucial for determining general or individual treatment strategies, as different histological subtypes require different treatment protocols [2,4,5].

Standard diagnostic methods for identifying NSCLC subtypes in clinical practice rely on histological or cytological examination of tumor samples obtained through biopsy [6]. However, biopsy is an invasive procedure that disrupts the tumor structure, increasing the risk of malignancy progression and metastasis [6]. Consequently, there is a growing need for non–invasive diagnostic methods to differentiate the histological subtypes of NSCLC. The non–invasive methods are primarily based on advanced imaging diagnostics – such as positron emission tomography (PET) combined with computed tomography (CT) or magnetic resonance imaging (MRI) [7–11] – assessed visually [12,13] or supported by advanced medical image analysis – including radiomics approaches [14–20]. Given that visual evaluation of lung cancer CT, MRI, PET/CT and PET/MRI image is subjective in nature, radiomics approaches are used for extraction and analysis lung cancer image features thus enhancing objectivity in diagnostic images evaluation. Among radiomics approaches, which uses data–characterization algorithms to extract a large number of features from medical images, texture analysis is most commonly applied to improve analysis of lung cancer diagnostic images [21]. Texture analysis evaluates the distribution and relationship of pixels in diagnostic images using structural methods (base on theory of mathematical morphology), model–based methods (such as fractal texture analysis and autoregressive model), statistical approaches (including first–order statistics (FOS) and second–order statistics (SOS)), or transformation methods (based on the Fourier, Gabor or Wavelet transform). Among them, the most commonly used texture features come from six main categories: (1) FOS; SOS including (2) Gradient, (3) Run–Length Matrix, and (4) Co–occurrence Matrix – all these representing statistical approaches; (5) autoregressive model – representing model–based methods; and (6) wavelets – representing transform methods [22]. FOS depicts the distribution of pixel values, Gradient measure the absolute gradient values of neighborhoods of pixels, Run–Length Matrix reflects the pixel intensity homogeneity in specific directions, Co–occurrence Matrix describes the relative relations of pixel pairs along all directions at different distances, while autoregressive model estimates the pixel intensity with four adjacent pixels [16]. And indeed, these texture features categories are implemented clinically for the analysis of lung cancer medical images supporting non–invasive differentiation of the NSCLC histological subtypes [14,16–18,20]. Tang et al. applied FOS and SOS – such as Gray–Level Co–occurrence Matrix (GLCM), Gray–Level Dependence Matrix (GLDM), Gray Level Run–Length Matrix (GLRLM), and Gray–Level Size Zone Matrix (GLSZM) to PET/MRI images [14]. Liu et al. applied FOS, SOS – such as Gradient, GLCM, and Run–Length Matrix (RLM) – as well as autoregressive model to CT images [16]. Ren et al. applied SOS – such as GLCM, GLRLM, GLSZM, Gray Level Neighborhood Intensity–Difference Matrix, and Neighboring Gray Level Dependence (NGLD) to PET/CT images [17]. Tang et al. applied FOS and SOS – such as GLCM, GLDM, GLRLM, GLSZM, and Neighboring Gray–Tone Difference Matrix (NGTDM) to PET/MRI and CT images [18]. Yang et al. applied FOS, SOS – such as GLCM, GLDM, GLRLM, GLSZM, and NGTDM – as well as Wavelet transform to MRI images [20]. All these researches provide more precise quantification of lung cancer heterogeneity using texture features than visual evaluation. Thus, the extracted texture features have been successfully applied in non–invasive NSCLC diagnosis and histological subtypes differentiation [14,16–18,20,21]. However, entropy–based texture features have not yet been effectively applied for this purpose.

In recent years, entropy has gained attention for the quantitative description of textures, especially in biomedical images [23–31]. Entropy–based methods represent statistical approaches to texture analysis. They utilize one–dimensional (1D) and two–dimensional (2D) data [32] returning five primary two–dimensional features measured at multiple scales: sample entropy (SampEn2D) [23], fuzzy entropy (FuzzEn2D) [24], permutation entropy (PermEn2D) [33], dispersion entropy (DispEn2D) [25], and distribution entropy (DistEn2D) [27]. Each entropy–based texture feature has specific properties; however, they share the advantage of directly calculating features on the image [31], yielding numerical results that reflect the irregularity or complexity of the analyzed diagnostic image [32]. Therefore, the motivation of this study arise from the implementation entropy–based methods to the non–invasive NSCLC diagnosis and histological subtypes differentiation, given the existing radiomics approaches provide image irregularity/complexity being particularly relevant for the quantification of lung cancer structure and consequently NSCLC subtypes differentiation [14,16–18,20,21].

Therefore, this study aimed to improve the efficiency of the computer–aided non–invasive diagnosis of NSCLC by supplementing the statistical approaches to MRI image texture analysis with entropy–based methods. The aim is achieved by completing the following steps: (1) identification of the regions of interest (ROIs) representing metabolically active lung tumors on PET/MRI images; (2) preprocessing of the MRI images using normalize filtration and median filtration; (3) extraction of the FOS, SOS, and entropy–based texture features from raw and filtered MRI images; (4) selection relevant FOS, SOS, and entropy–based texture feature for NSCLC subtypes classification; (5) utilization of the selected FOS, SOS, and entropy–based texture features for ADC and SCC subtypes classification using three algorithms; (6) evaluation of the classification performance of each used dataset; and (7) select the highest efficiency combination of filtration, texture features, and classification algorithm used for computer–aided diagnosis of NSCLC subtypes.

## 2 Materials and methods

### 2.1 Data collection

The study analyzed 154 MRI images from 31 patients (20 men (65%) and 11 women (35%); mean age: 65 years, range: 35–82 years) with metabolically active lung tumors confirmed through PET/MRI. The data were collected by the Independent Laboratory of Molecular Imaging at the Medical University of Bialystok between January 15, 2020, and March 31, 2022 in accordance with the permission (No R I 002/94/2018) of the Local Ethical Committee at the Medical University of Bialystok in Poland. Informed written consent was obtained from all study participants. Scans were conducted using the Magnetom Biograph mMR (Siemens Healthcare, Erlangen, Germany) with a contrast–enhancing protocol and the following parameters: transverse breathing T1_VIBE sequence, TR 4.02 ms, TE 1.6 ms, slice thickness 4 mm, field of view (FoV) 400 mm, matrix size 256, and GRAPPA factor 2.

**Fig 1:**
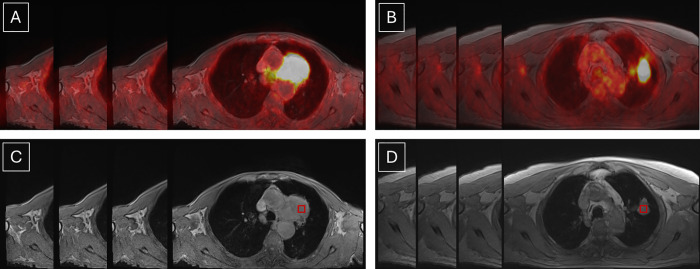
Images and selected regions of interest (ROIs) from analyzes scans of metabolically active lung tumors. ROIs were annotated as red squares. (A) PET/MRI images of adenocarcinoma (ADC), (B) PET/MRI images of squamous cell carcinoma (SCC), (C) MRI images of ADC with annotated ROI, (D) MRI images of SCC with annotated ROI.

Histopathological examination confirmed ADC in 12 patients (39%) and SCC in 19 patients (61%). Consequently, 75 PET/MRI images of ADC and 79 PET/MRI images of SCC were analyzed (Fig 1). PET/MRI images were assessed by two independent radiology specialists (M.M. and M.H.), so that the structural and metabolic features of NSCLC were annotated in each case. Any disagreement were solved by the third part (M.D.), so that the final tumor location, size, and metabolic activity were unified for each NSCLC. Tumors were located in both the upper and lower lung lobes, exhibiting a range of sizes and degrees of invasiveness. The mean size of ADC tumors was 38 mm (range: 17–90 mm), while SCC tumors averaged 49 mm (range: 24–79 mm). The maximum Standardized Uptake Value (SUVmax) (mean ± SD) was 8.03 ± 4.06 for ADC and 11.86 ± 3.42 for SCC, with all tumors displaying SUVmax > 3.54, confirming their metabolic activity.

### 2.2 ROIs segmentation and processing

PET/MRI images were used to identify metabolically active areas of the affected lung. On the corresponding MRI images, ROIs were manually annotated by one researcher (E.B.) within these metabolically active areas. The masks, each measuring 128 x 128 pixels, were annotated using the ImageJ software (Wayne Rasband, National Institutes of Health, USA), and ROIs were saved as BMP files.

**Fig 2:**
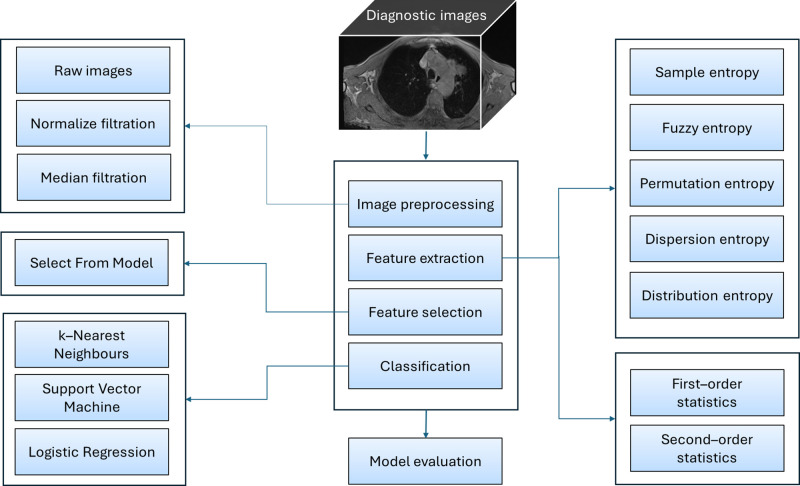
The workflow of processing and analysis of regions of interest (ROIs) annotated on MRI images of lung adenocarcinoma (ADC) and squamous cell carcinoma (SCC). The protocol included image processing, feature extraction, feature selection, and classification. The classification performance of the protocol was then evaluated.

The ROIs were subsequently processed and analyzed according to the workflow presented in Fig 2. The protocol included four main steps: image processing, feature extraction, feature selection, and classification. On the image processing step, ROIs were filtered using a normalize and median filtration to enhance image quality. On the feature extraction step, FOS texture features, SOS texture features (using GLCM, GLDM, GLRLM, GLSZM, and NGTDM methods), and two–dimensional entropy–based texture features (SampEn2D, FuzzEn2D, PermEn2D, DispEn2D, DistEn2D) were calculated. Entropy–based texture features were calculated at five scales. Each texture feature was calculated for raw images (unfiltered), and both filtered ROIs. On the feature selection step, the extracted features underwent statistical analysis to identify significant differences between NSCLC histological subtypes. Feature selection was then performed using Select From Model (SFM) protocol. On the classification step, machine learning (ML) models – including k–Nearest Neighbors (kNN), Support Vector Machines (SVM), and Logistic Regression (LR) – were employed to classify MRI images as representing ADC or SCC. Finally, model evaluation metrics such as accuracy, sensitivity, specificity, precision, F1 index, and Area Under Curve (AUC) were calculated to assess classification performance.

### 2.3 Image processing

The following two filtering algorithms were used to reduce the noise in the MRI images. The following filtering methods were implemented in SimpleITK (https://simpleitk.org/) toolkit in Python [34]:

(i) *Normalize filtration* – a linear filtration in which the output image is a rescaled image in which the pixels have zero mean and unit variance;
(ii) *Median filtration* – a non–linear filtration in which the output image pixels are the median of the pixels in the neighborhood of the input pixel being calculated.

### 2.4 Feature extraction

The following texture analysis approaches were used to extract texture features of the MRI image from each ROI, separately. FOS and SOS texture features were calculated using the PyRadiomics package version 3.1.0 in Python. Entropy–based texture features were calculated using EntropyHub (https://www.entropyhub.xyz/), an open–source Python package for the extraction of entropy features from MRI images [35].

#### 2.4.1 First–order statistics.

The FOS approach yielded 17 texture features. Details of FOS texture features are described in our previous, related study [36].

#### 2.4.2 Second–order statistics.

The SOS approach yielded a total of 73 texture features, extracted using five methods: GLCM, GLDM, GLRLM, GLSZM, and NGTDM methods. GLCM yielded 22 texture features, GLDM yielded 14 texture features, GLRLM yielded 16 texture features, GLSZM yielded 16 texture features, while NGTDM yielded 5 texture features. Details of SOS texture features are described in our previous, related study [36].

#### 2.4.3 Multiscale entropy.

An algorithm for calculating two–dimensional multiscale entropy quantifies the complexity of an image, where it is defined as a measure of irregularity at several spatial scales [31,37]. Multiscale measures are based on complexity and consist of two main steps: a coarse–graining process, which involves removing high–frequency image components using a digital low–pass filter, and down–sampling the filtered data by a scale factor s, and calculating the entropy method for all coarse–grained data at each scale s. The multiscale approach can be used as an extension of SampEn2D, FuzzEn2D, PermEn2D, DispEn2D, or DistEn2D. A decrease in entropy values at spatial scales indicates that the image may be irregular but not structurally complex. However, when no noticeable changes in entropy values are observed at different scales, this suggests that the image retains complex structures at multiple scale factors; the image is said to be complex [38]. The following five entropy–based methods in five scales yielded a total of 25 texture features.

**Two–dimensional sample entropy.** SampEn2D of images is an extension of the one–dimensional time series measure of sample entropy [39]. The SampEn2D method defines two–dimensional patterns of length m. Each window of length m is then compared to all other windows of length m in the image. Pattern matching refers to the case where each pixel in one window differs no more than r from the corresponding pixel in the window with which it is compared. The average occurrence probability is calculated for all windows of length m and m + 1. SampEn2D is defined as the logarithm of the calculated average probabilities. The regular images or periodic structures in images have the same number of patterns for both m and m + 1. Therefore, regular images achieve low SampEn2D values, while irregular images return high SampEn2D values.

**Two–dimensional fuzzy entropy.** FuzzEn2D is an extension of the one–dimensional measure of fuzzy entropy [29]. FuzzEn2D is defined as the negative natural logarithm of the conditional probability that two patterns similar for their corresponding m points will remain similar for the next m + 1 points. It uses a continuous exponential function to determine the degree of similarity between vectors. Images containing regular patterns or periodic structures have a low FuzzEn2D value, while images with irregular patterns or non–periodic structures have a high FuzzEn2D value.

**Two–dimensional permutation entropy.** PermEn2D is an extension of one–dimensional permutation entropy [33]. The PermEn2D method defines two–dimensional patterns of length m. From each window of size m, an ordered vector representing the pixel intensities is created, which is encoded by permutations. PermEn2D is the Shannon entropy of the distribution of order patterns in the image. The normalized value of PermEn2D takes values in the range <0,...,1 > , meaning that a value of 1 specifies random data, while a value smaller than 1 indicates some kind of correlated dynamics. PermEn, although simple and computationally fast, does not take into account the mean value of amplitudes and differences between amplitude values. Images containing regular patterns have a low PermEn2D value, while images with irregular patterns have a high PermEn2D value.

**Two–dimensional dispersion entropy.** DispEn2D entropy is an extension of one–dimensional dispersion entropy [25]. The idea of calculation DispEn2D relies on mapping to c classes the values of image pixels. After mapping, the obtained results are matched to the dispersion pattern π, and the probabilities p(π) of each dispersion patterns are calculated. If all possible, two–dimensional image dispersion patterns have the same probability value, DispEn2D reaches its maximum value. If there is one probability value p other than zero, DispEn2D reaches the minimum value. Images containing regular patterns have a low DispEn2D value, while images with irregular patterns have a high DispEn2D value.

**Two–dimensional distribution entropy.** DistEn2D was introduced to the quantitative description of the irregularities of the images, taking into account the small size of the image [27]. In the process of counting the amount of similarity between two patterns, the distance between the corresponding windows is measured. The histogram of the distance matrix is used to estimate the empirical probability density function (ePDF). Regular images return low DistEn2D values, while irregular images return high DistEn2D values.

### 2.5 Statistical analysis

Statistical analysis was performed using the Python library scipy (https://scipy.org/) [40] only on the entropy–based methods dataset containing 25 texture features. Data were presented independently as data series for raw images and two filtrations. Data series were tested separately for univariate distributions using a Shapiro–Wilk normality test. Data analysis was performed in the following two steps: (i) testing the differences between data series of the ADC and SCC groups; (ii) calculating the coefficient of variation for the consecutive features in each texture analysis approach, including both filters used. The comparisons between (i) data series representing ADC and SCC groups were assessed using the t–test for data with normal distribution and the U Mann–Whitney test for data that did not have normal distribution. The alpha value was established as α = 0.05.

### 2.6 Feature selection

Input data containing features were standardized using the StandardScaler from the Scikit–learn library in Python version 3.11 (https://scikit–learn.org/stable/index.html accessed on 2 June 2025) [41]. Feature selection was performed using SFM on three datasets: (1) statistical methods dataset (containing 17 FOS texture features and 73 SOS texture features), (2) entropy–based methods dataset (containing 25 texture features), and (3) combined statistical and entropy–based methods dataset (containing 17 FOS texture features, 73 SOS texture features, and 25 texture features). In each dataset, SFM selected the 25 features with the highest feature importance. This parameter was selected experimentally to obtain the highest accuracy of the final ML–based classification.

Given that SFM is based on the random forest method, the feature selection process involved fitting a random forest model to the data and assessing the significance of individual features using the Python library Scikit–learn [41]. A random forest model with 100 estimators (decision trees) was used to assess the significance of features. The algorithm was calibrated using a random seed (random_state = 42) to make the results replicable. Once the model was trained, each feature was scored using a measure of feature importance (feature importance), which reflected its impact on the classification score. The classes in the sklearn.feature_selection (https://scikit–learn.org/) [41] module were used for the selection of features/dimension reduction on sample sets, both to improve the accuracy results of estimators and to increase their performance on multidimensional data sets.

### 2.7 Classification

The ML models allow the assignment of the correct diagnosis based on the attributes of the input images. The following three classification methods were used.

#### 2.7.1 k–nearest neighbours.

The kNN algorithm is one of the simplest and most widely used classification algorithms for medical image analysis. Its operation is based on the simple hypothesis that objects close to each other in functional space belong to the same class. In classification, the kNN algorithm assigns a new point to the class that dominates among its nearest neighbours k in the feature space [42]. The kNN classification steps were executed with predefined hyperparam–eters: k = 7, using the Euclidean distance metric and uniform weighting (https://scikit–learn.org accessed on 2 June 2025).

#### 2.7.2 Support vector machines.

The SVM is a classification algorithm that is commonly used for classification and regression tasks, especially when the data is complex and multidimensional. The basic idea of SVM is to find a hyperplane in the characteristic space that minimizes the separation of data belonging to different classes. The SVM algorithm aims to maximize the margin, i.e., the distance between the nearest data points (called support vectors) and the decision–making hyperplane. A high value of this margin allows for a better generalization of the model and a lower risk of over–alignment [43]. The SVM classification steps were executed with predefined hyperparameters: a cost parameter C = 100, and a radial basis function (RBF) kernel with gamma = 0.0001; numerical tolerance was set to 0.001 (https://scikit–learn.org accessed on 2 June 2025).

#### 2.7.3 Logistic regression.

The LR is a regression method used to predict the value of the dependent variable. When creating an LR equation, maximum likelihood ratios are used to determine the statistical significance of the variables. LR is useful in situations where the presence or absence of a trait or outcome needs to be predicted from the values of a set of predictor variables [44]. The LR classification steps were executed with predefined hyperparameters: tolerance for stopping criteria tol = 0.0001, inverse regularization strength C = 1.0, with the Broyden–Fletcher–Goldfarb–Shanno optimization algorithm, and regularization with L2 norm (https://scikit–learn.org accessed on 2 June 2025).

### 2.8 Model evaluation metrics

The following metrics were evaluated for each dataset combined with each classification algorithm: accuracy, precision, recall, and F1 index. Model evaluation metrics were calculated using the following formulas:

- accuracy:

$$Accuracy=\frac{TP+TN}{TP+TN+FP+FN}$$

- precision:

$$Precision=\frac{TP}{TP+FP}$$

- recall:

$$Recall=\frac{TP}{TP+FN}$$

- F1:

$$F\text{1}=\text{2}\cdot \frac{Precision\cdot recall}{Precision+recall}$$

where: true positive (TP) is the number of correctly classified examples from the selected class; false negative (FN) is the number of incorrectly classified examples from this class (a negative decision when it should be positive); true negative (TN) is the number of correctly not assigned to the selected class; and false positive (FP) is the number incorrectly assigned to the selected class when in fact they do not belong to it [45,46].

Additionally, the AUC represented area under the ROC curve, which shows the relationship between recall and false positive rate (FPR) calculated using the following formula:


$$FPR=\frac{FP}{FP+TN}$$

Given that, the classification performance was evaluated using 5–fold stratified cross–validation, the results are presented in tables as mean ± standard deviation (SD) from 5–folds cross–validation.

## 3 Results

### 3.1 Entropy–based texture features

**Fig 3:**
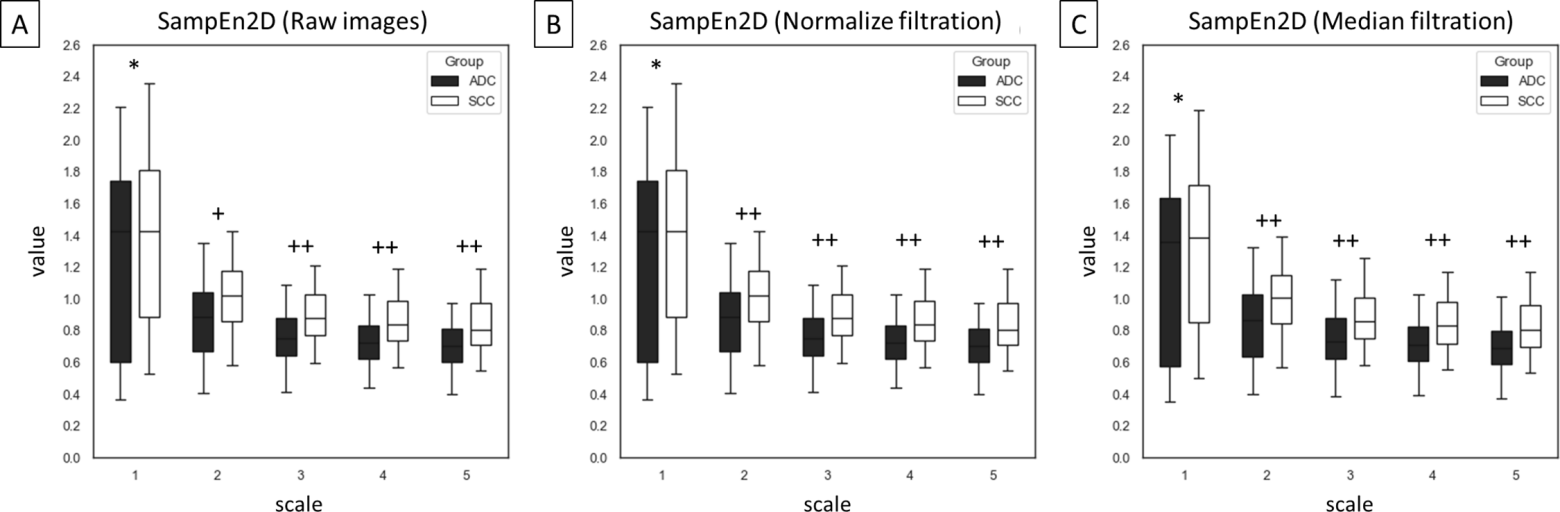
Two–dimensional sample entropy (SampEn2D) measured in five scales and compared between adenocarcinoma (ADC) and squamous cell carcinoma (SCC) groups for (A) raw mages, (B) normalize filtration, and (C) median filtration. Data are presented using minimum and maximum values, lower and upper quartiles, and median. Differences between ADC and SCC groups were marked with * for p < 0.05; ** for p < 0.01; + for p < 0.001; and ++ for p < 0.0001. Differences were considered significant for p < 0.05.

**Fig 4:**
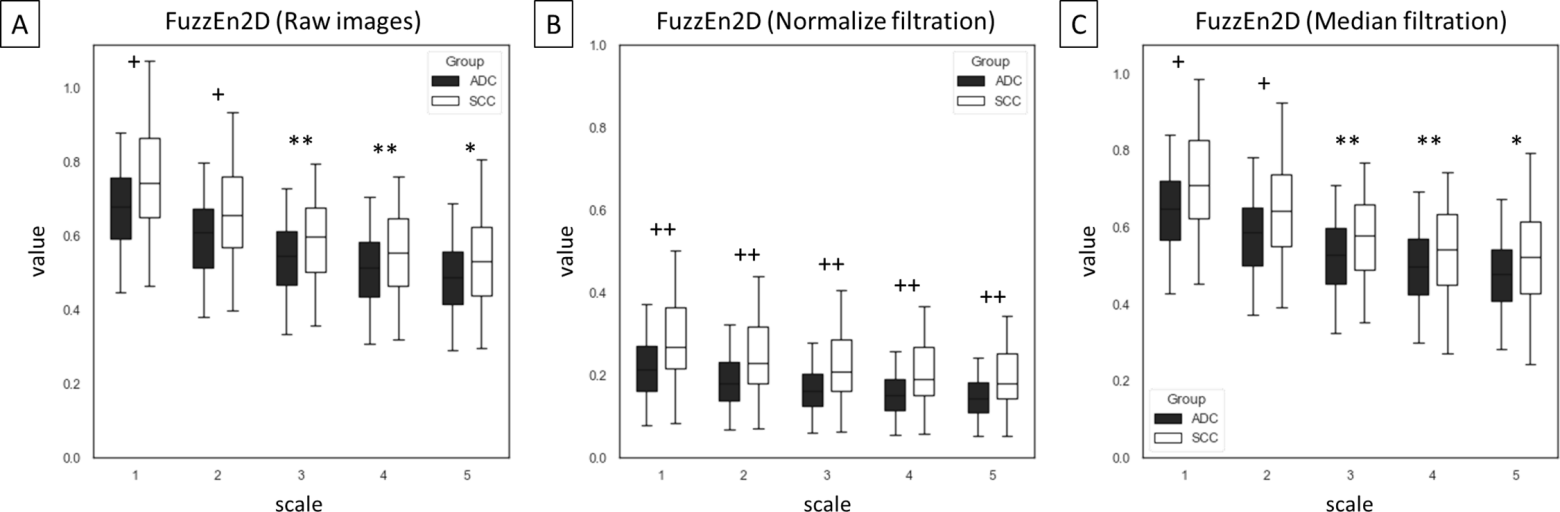
Two–dimensional fuzzy entropy (FuzzEn2D) measured in five scales and compared between adenocarcinoma (ADC) and squamous cell carcinoma (SCC) groups for (A) raw images, (B) normalize filtration, and (C) median filtration. Data are presented using minimum and maximum values, lower and upper quartiles, and median. Differences between ADC and SCC groups were marked with * for p < 0.05; ** for p < 0.01; + for p < 0.001; and ++ for p < 0.0001. Differences were considered significant for p < 0.05.

**Fig 5:**
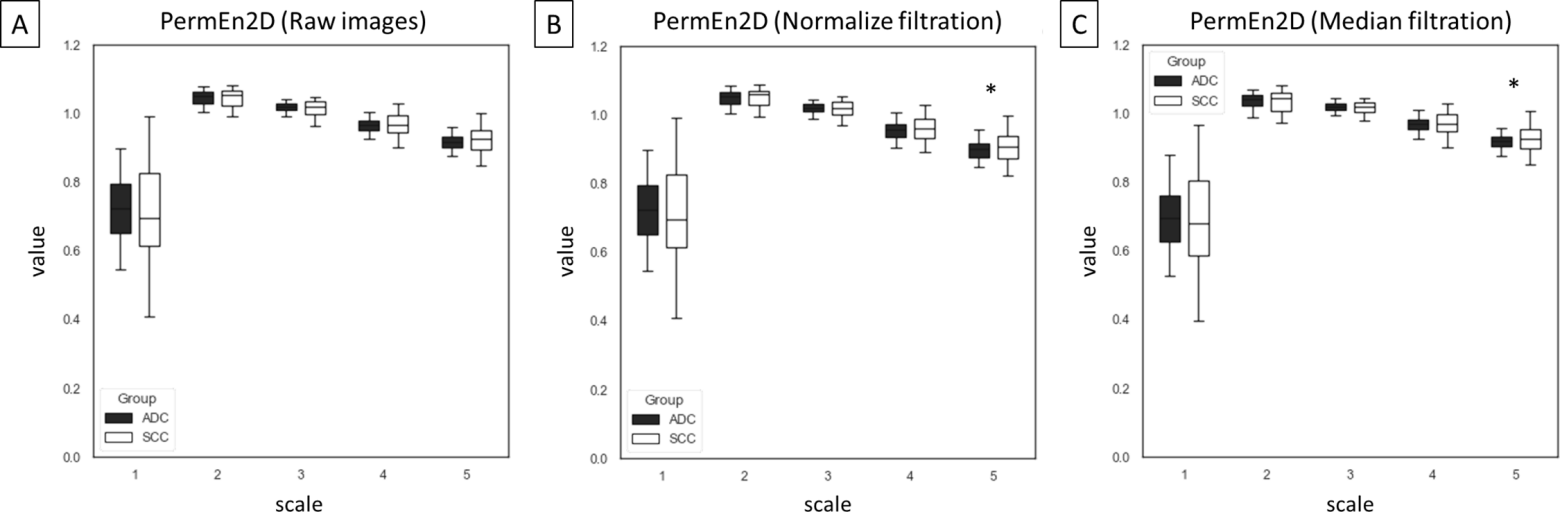
Two–dimensional permutation entropy (PermEn2D) measured in five scales and compared between adenocarcinoma (ADC) and squamous cell carcinoma (SCC) groups for (A) raw images, (B) normalize filtration, and (C) median filtration. Data are presented using minimum and maximum values, lower and upper quartiles, and median. Differences between ADC and SCC groups were marked with * for p < 0.05; ** for p < 0.01; + for p < 0.001; and ++ for p < 0.0001. Differences were considered significant for p < 0.05.

**Fig 6:**
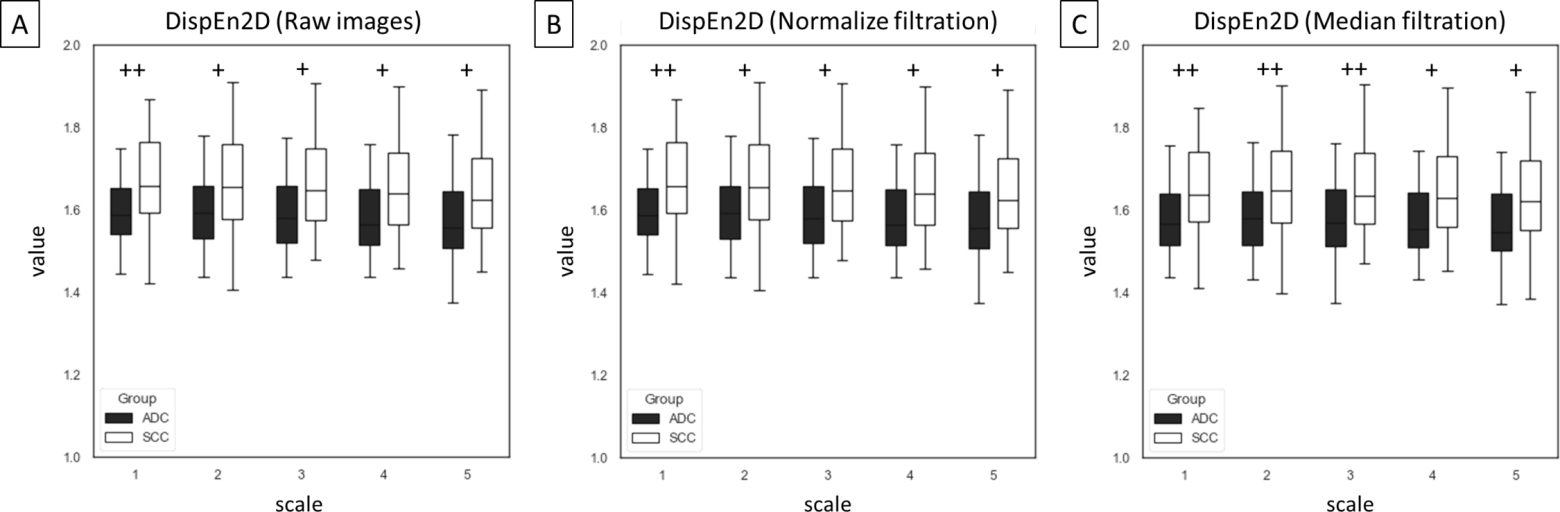
Two–dimensional dispersion entropy (DispEn2D) measured in five scales and compared between adenocarcinoma (ADC) and squamous cell carcinoma (SCC) groups for (A) raw images, (B) normalize filtration, and (C) median filtration. Data are presented using minimum and maximum values, lower and upper quartiles, and median. Differences between ADC and SCC groups were marked with * for p < 0.05; ** for p < 0.01; + for p < 0.001; and ++ for p < 0.0001. Differences were considered significant for p < 0.05.

**Fig 7:**
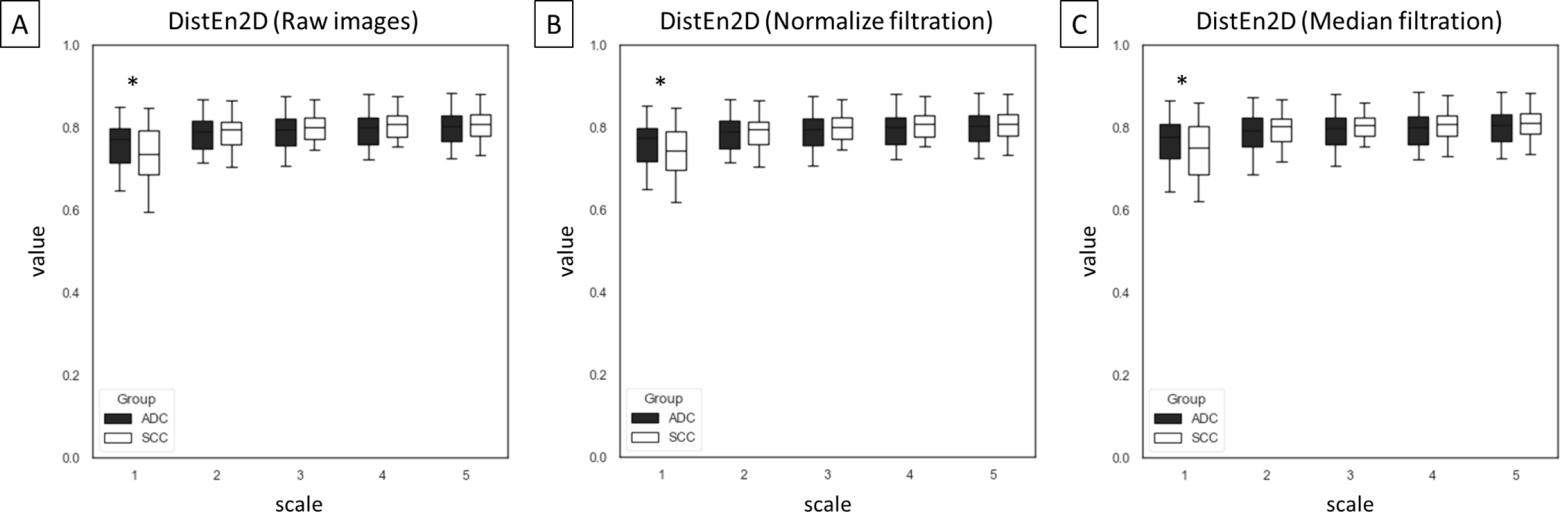
Two–dimensional distribution entropy (DistEn2D) measured in five scales and compared between adenocarcinoma (ADC) and squamous cell carcinoma (SCC) groups for (A) raw images, (B) normalize filtration, and (C) median filtration. Data are presented using minimum and maximum values, lower and upper quartiles, and median. Differences between ADC and SCC groups were marked with * for p < 0.05; ** for p < 0.01; + for p < 0.001; and ++ for p < 0.0001. Differences were considered significant for p < 0.05.

Among 75 entropy–based texture features, returned from combinations of five entropy–based methods (n = 5), five scales (n = 5), and three image types – raw images and two filtrations (n = 3), differences between ADC and SCC groups were presented for SampEn2D (Fig 3), FuzzEn2D (Fig 4), PermEn2D (Fig 5), DispEn2D (Fig 6), and DistEn2D (Fig 7), separately. SampEn2D was higher in SCC than in ADC on each scale and image type. However, the significance level (p < 0.0001) for scales 3–5 on raw images, as well as scales 2–5 on both filtered images, was the lowest (Fig 3). FuzzEn2D was higher in SCC than in ADC on each scale and image type. However, the significance level was the lowest (p < 0.0001) for all scales on normalize filtered images (Fig 4). PermEn2D was higher in SCC than in ADC only on scale 5 on filtered images (Fig 5). DispEn2D was higher in SCC than in ADC on each scale and image type. However, the significance level (p < 0.0001) was the lowest for scale 1 on raw images, scale 1 on normalize filtered images, and scales 1–3 on median filtered images (Fig 6). PermEn2D differed between SCC and ADC only on scale 1, however, on all image types (Fig 7).

### 3.2 Feature selection

**Fig 8:**
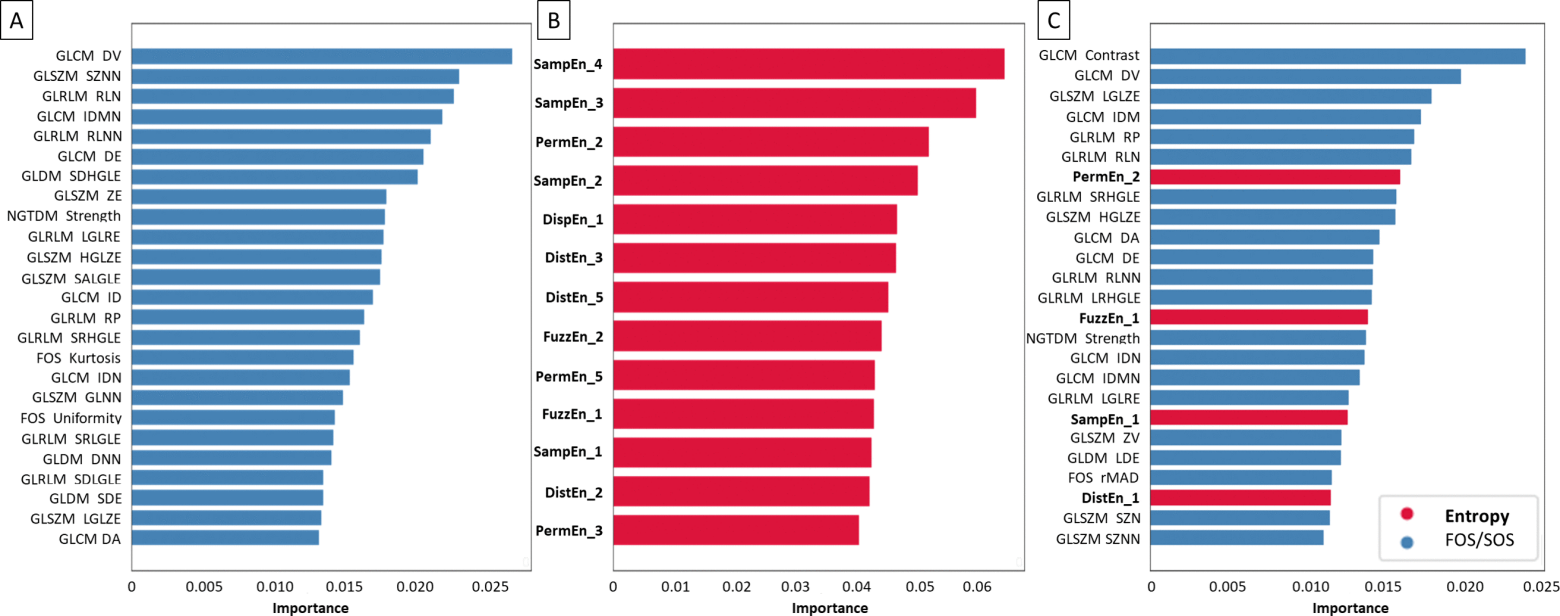
Coefficient of importance for selected (A) FOS and SOS texture features, (B) entropy–based texture features, (C) combined FOS, SOS, and entropy–based texture features extracted from unfiltered raw images. FOS and SOS texture features are marked by blue bars, while entropy–based texture features are marked by red bars and bold font.

Considering raw images, the following texture features were established as most important for NSCLC histological subtypes classification. Within the FOS/SOS dataset, predominately specific GLCM, GLSZM, GLRLM, and GLDM features were selected (Fig 8A). Within the entropy–based dataset, predominately SampEn2D (on scales 4 > 3 > 2 > 1), PermEn2D (on scales 2 > 5 > 3), DistEn2D (on scales 3 > 5 > 2), and FuzzEn2D (on scales 2 > 1) were selected (Fig 8B). Within the combined dataset, among mainly GLCM, GLSZM, and GLRLM features, PermEn2D (on scale 2), FuzzEn2D (on scale 1), SampEn2D (on scale 1), and DistEn2D (on scale 1) were selected (Fig 8C).

**Fig 9:**
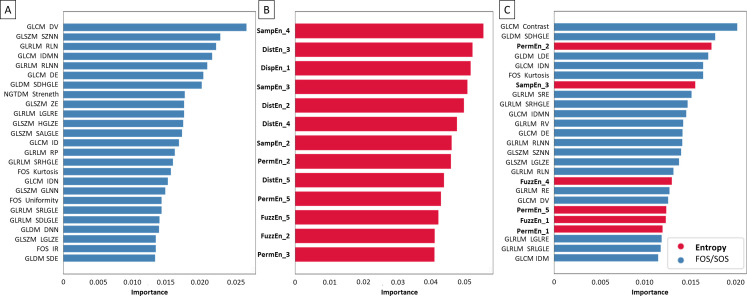
Coefficient of importance for selected (A) FOS and SOS texture features, (B) entropy–based texture features, (C) combined FOS, SOS, and entropy–based texture features extracted from normalize filtered images. FOS and SOS texture features are marked by blue bars, while entropy–based texture features are marked by red bars and bold font.

Considering normalize filtered images, the following texture features were established as most important for NSCLC histological subtypes classification. Within the FOS/SOS dataset, predominately specific GLCM, GLSZM, GLRLM, and GLDM features were selected (Fig 9A). Within the entropy–based dataset, predominately SampEn2D (on scales 4 > 3 > 2), DistEn2D (on scales 3 > 2 > 4 > 5), PermEn2D (on scales 2 > 5 > 3), and FuzzEn2D (on scales 5 > 2) were selected (Fig 9B). Within the combined dataset, among mainly GLCM features, GLDM features, PermEn2D (on scales 2 > 5 > 1), FOS features, SampEn2D (on scale 3), GLRLM features, GLSZM features, FuzzEn2D (on scales 4 > 1) were selected (Fig 9C).

**Fig 10:**
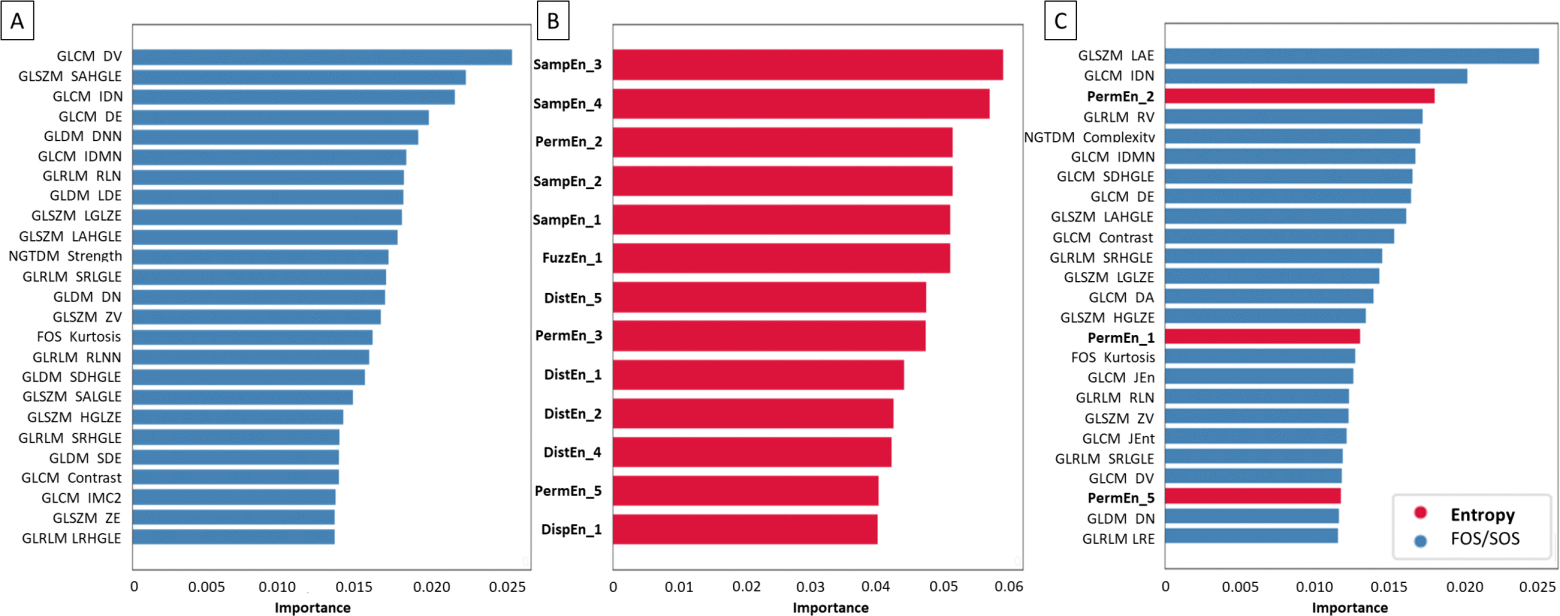
Coefficient of importance for selected (A) FOS and SOS texture features, (B) entropy–based texture features, (C) combined FOS, SOS, and entropy–based texture features extracted from median filtered images. FOS and SOS texture features are marked by blue bars, while entropy–based texture features are marked by red bars and bold font.

Considering median filtered images, the following texture features were established as most important for NSCLC histological subtypes classification. Within the FOS/SOS dataset, predominately specific GLCM, GLSZM, GLDM, NGTDM, and GLRLM features were selected (Fig 10A). Within the entropy–based dataset, predominately SampEn2D (on scales 3 > 4 > 2 > 1), PermEn2D (on scales 2 > 3 > 5), and DistEn2D (on scales 5 > 1 > 2 > 4) were selected (Fig 10B). Within the combined dataset, among mainly GLSZM features, GLCM features, PermEn2D (on scales 2 > 1 > 5), GLRLM features, NGTDM features, and GLSZM features were selected (Fig 10C).

### 3.3 Classification

To evaluate the performance of the statistical and entropy–based ML–supported classification of the studied histological subtypes of NSCLC, the model evaluation metrics and the AUC values for the ROC curves are summarized in Tables 1, 2, 3. Among the FOS and SOS texture features, the kNN model achieved the highest accuracy, precision, and AUC for MRI images after median filtration as well as the highest recall and F1 index for unfiltered MRI images and images after normalize filtration. After median filtration, the SVM model achieved the highest evaluation metrics for MRI images. The LR model achieved the highest accuracy, precision, recall, and F1 index for MRI images after median filtration as well as the highest AUC for unfiltered MRI images and images after normalize filtration. When comparing the ML models, the SVM model achieved the highest evaluation metrics (Table 1).

**Table 1:** Model evaluation metrics (mean ± SD from 5–folds cross–validation) for classification models (kNN, SVM, LR) build on selected FOS and SOS texture features for unfiltered raw images, normalize filtration, and median filtration. The highest value of each metric for each classification model is marked in bold font.

Classification		Filtration		
Models	Metrics	Unfiltered raw images	Normalize filtration	Median filtration
**kNN**	Accuracy	0.62 ± 0.05	0.62 ± 0.06	**0.65 ± 0.04**
	Precision	0.64 ± 0.05	0.64 ± 0.06	**0.69 ± 0.06**
	Recall	**0.62 ± 0.12**	**0.62 ± 0.09**	0.58 ± 0.11
	F1	**0.63 ± 0.07**	**0.63 ± 0.06**	0.62 ± 0.08
	AUC	0.71 ± 0.04	0.71 ± 0.04	**0.72 ± 0.03**
**SVM**	Accuracy	0.67 ± 0.07	0.67 ± 0.06	**0.70 ± 0.03**
	Precision	0.71 ± 0.08	0.71 ± 0.08	**0.75 ± 0.07**
	Recall	0.61 ± 0.08	0.61 ± 0.06	**0.62 ± 0.04**
	F1	0.66 ± 0.07	0.66 ± 0.06	**0.68 ± 0.01**
	AUC	0.74 ± 0.04	0.74 ± 0.04	**0.75 ± 0.03**
**LR**	Accuracy	0.64 ± 0.05	0.64 ± 0.05	**0.67 ± 0.07**
	Precision	0.65 ± 0.07	0.65 ± 0.06	**0.70 ± 0.09**
	Recall	0.63 ± 0.07	0.63 ± 0.05	**0.65 ± 0.04**
	F1	0.64 ± 0.05	0.64 ± 0.04	**0.67 ± 0.05**
	AUC	**0.75 ± 0.03**	**0.75 ± 0.03**	0.73 ± 0.08

Notes: FOS – first–order statistics, SOS – second order statistics, kNN – k–Nearest Neighbours, SVM – Support Vector Machines, LR – Logistic Regression.

**Table 2:** Model evaluation metrics (mean ± SD from 5–folds cross–validation) and Area Under Curve (AUC) for classification models (kNN, SVM, LR) build on selected entropy–based texture features for unfiltered raw images, normalize filtration, and median filtration. The highest value of each metric for each classification model is marked in bold font.

Classification		Filtration		
Models	Metrics	Unfiltered raw images	Normalize filtration	Median filtration
**kNN**	Accuracy	0.64 ± 0.06	0.67 ± 0.07	**0.68 ± 0.05**
	Precision	0.67 ± 0.07	0.69 ± 0.06	**0.70 ± 0.05**
	Recall	0.60 ± 0.14	0.64 ± 0.14	**0.67 ± 0.10**
	F1	0.62 ± 0.09	0.66 ± 0.09	**0.68 ± 0.06**
	AUC	0.69 ± 0.07	0.73 ± 0.04	**0.75 ± 0.06**
**SVM**	Accuracy	0.66 ± 0.07	**0.70 ± 0.09**	0.69 ± 0.09
	Precision	0.72 ± 0.11	**0.75 ± 0.12**	0.71 ± 0.11
	Recall	0.60 ± 0.06	0.62 ± 0.07	**0.67 ± 0.11**
	F1	0.65 ± 0.06	0.68 ± 0.08	**0.69 ± 0.09**
	AUC	**0.75 ± 0.06**	**0.75 ± 0.07**	**0.75 ± 0.06**
**LR**	Accuracy	0.64 ± 0.08	0.67 ± 0.09	**0.68 ± 0.09**
	Precision	0.67 ± 0.08	**0.70 ± 0.11**	**0.70 ± 0.10**
	Recall	0.65 ± 0.11	0.65 ± 0.10	**0.67 ± 0.14**
	F1	0.65 ± 0.09	0.67 ± 0.09	**0.68 ± 0.10**
	AUC	**0.73 ± 0.07**	**0.73 ± 0.07**	**0.73 ± 0.07**

Notes: kNN – k–Nearest Neighbours, SVM – Support Vector Machines, LR – Logistic Regression.

**Table 3:** Model evaluation metrics (mean ± SD from 5–folds cross–validation) and Area Under Curve (AUC) for classification models (kNN, SVM, LR) build on selected FOS, SOS, and entropy–based texture features for unfiltered raw images, normalize filtration, and median filtration. The highest value of each metric for each classification model is marked in bold font.

Classification		Filtration		
Models	Metrics	Unfiltered raw images	Normalize filtration	Median filtration
**kNN**	Accuracy	0.65 ± 0.05	0.64 ± 0.07	**0.70 ± 0.04**
	Precision	0.66 ± 0.06	0.65 ± 0.08	**0.77 ± 0.05**
	Recall	**0.65 ± 0.09**	0.63 ± 0.05	0.58 ± 0.07
	F1	0.65 ± 0.05	0.64 ± 0.06	**0.66 ± 0.06**
	AUC	0.72 ± 0.06	0.71 ± 0.04	**0.76 ± 0.06**
**SVM**	Accuracy	0.66 ± 0.05	0.67 ± 0.05	**0.71 ± 0.06**
	Precision	0.70 ± 0.07	0.71 ± 0.07	**0.77 ± 0.07**
	Recall	0.61 ± 0.05	0.61 ± 0.06	**0.62 ± 0.06**
	F1	0.65 ± 0.05	0.65 ± 0.05	**0.69 ± 0.05**
	AUC	0.73 ± 0.04	0.75 ± 0.06	**0.79 ± 0.05**
**LR**	Accuracy	0.68 ± 0.04	0.68 ± 0.05	**0.75 ± 0.05**
	Precision	0.68 ± 0.04	0.71 ± 0.05	**0.78 ± 0.04**
	Recall	0.72 ± 0.07	0.67 ± 0.07	**0.73 ± 0.08**
	F1	0.70 ± 0.05	0.68 ± 0.05	**0.75 ± 0.06**
	AUC	0.75 ± 0.05	0.75 ± 0.04	**0.80 ± 0.07**

Notes: FOS – first–order statistics, SOS – second order statistics, kNN – k–Nearest Neighbours, SVM – Support Vector Machines, LR – Logistic Regression.

Among the entropy–based texture features, the kNN model achieved the highest evaluation metrics for MRI images after median filtration. The SVM model achieved the highest accuracy and precision for MRI images after normalize filtration as well as the highest recall and F1 index for MRI images after median filtration with the same AUC for all image types. The LR model achieved the highest accuracy, precision, recall, and F1 index for MRI images after median filtration as well as the same AUC for all image types. When comparing the ML models, the SVM model achieved the highest evaluation metrics (Table 2).

Among the combined FOS, SOS, and entropy–based texture features, the kNN model achieved the highest accuracy, precision, F1 index, and AUC for MRI images after median filtration as well as the highest recall for unfiltered MRI images. After median filtration, the SVM and LR models achieved the highest evaluation metrics for MRI images. When comparing the ML models, the LR model achieved the highest evaluation metrics (Table 3). Moreover, among all models studied, just the LR model – worked on the combined FOS, SOS, and entropy–based texture features – achieved the highest evaluation metrics for MRI images after median filtration.

## 4 Discussion

The proposed protocol for MRI image segmentation, processing, and analysis facilitates the extraction of entropy–based texture features from lung cancer patients. This study employed PET/MRI images to confirm tumor metabolic activity, while feature extraction and analysis were conducted solely on MRI images. A similar approach was previously applied on PET/MRI [14,18,36,47] and PET/CT [17] images, suggesting that PET–based ROI identification is valid for subsequent image texture analysis. On the other hand, such an ROI placement, which relied on PET/MRI images, may limit generalizability to MRI–only segmentation and could introduce selection bias. Considering this limitation, further studies are needed to test the extraction of entropy–based texture features on MRI–only localization, as it was done to test the extraction of FOS and SOS texture features [20], or by using automated MRI image segmentation.

In the previous studies, texture features were extracted using FOS [14,18,36,47] and SOS [14,17,36,47], including GLCM [14,17,18,36,47], GLDM [14,18,36], GLRLM [14,17,36,47], GLSZM [14,17,18,36], NGLD [17], NGTDM [18,36], and Gradients [47]; however, not by using entropy–based texture features. Moreover, non–PET–based studies that extracted texture features from CT [16] and MRI [20] images do not use an entropy–based approach. In these studies, texture features were also extracted using FOS [16,20] and SOS [16,20], including GLCM [16,20], RLM [16], GLDM [20], GLRLM [20], GLSZM [20], NGTDM [20], and Gradients [16]. Therefore, this study may be considered the first application of entropy–based methods for computer–aided diagnosis of NSCLC subtypes.

Moreover, the previous studies have not used any image pre–processing [16,17,20] or have used normalize/Gaussian filtering [14,18,36,47] for image pre–processing; However, none of them compare extracted features between raw and filtered images as well as between filtering methods. In this study, only images filtered with both methods (normalize and median filtration) showed significant differences in PermEn2D between ADC and SCC groups. At the same time, for SampEn2D, FuzzEn2D, and DispEn2D at specific scales, lower significance levels were obtained for filtered images than for raw images. Since PermEn2D obtained the highest importance of features selected for classification, and the same ML algorithms achieved higher performance for images after filtering than for raw images, incorporating image pre–processing into the applied protocol seems advisable. One may observe that normalize filtration is a linear filtration used to reduce Gaussian noise produced by the variations in image intensity drawn from a Gaussian normal distribution. This kind of filtration increases contrast [48]. On the other hand, the median filtration is a non–linear filtration used to reduce salt and pepper noise produced by random occurrences of black and white pixels. This kind of filtration increases contrast with edge–preserving quality [48,49]. Since the same ML algorithms achieved higher performance for images after median filtration than normalize filtration, salt and pepper noise reduction may be more important for processing MRI images of NSCLC than Gaussian noise reduction.

This study found that MRI images of lung ADC exhibited more regular patterns or periodic structures, as reflected in lower values of SampEn2D, FuzzEn2D, and DispEn2D across five scales, as well as lower values of DistEn2D in scale 1 and PermEn2D in scale 5. In contrast, MRI images of lung SCC showed more irregular patterns or less periodic structures, indicated by higher corresponding entropy values. As noted in the paragraph above, differences in SampEn2D, FuzzEn2D, DispEn2D, and DistEn2D were detectable in all studied image types; however, differences in PermEn2D were statistically detectable only in images after normalize and median filtration. Despite the statistical differences of PermEn2D between ADC and SCC groups appearing weak, incorporating the PermEn2D into the conventional statistical radiomic features improved the classification metrics of computer–aided diagnosis of NSCLC histological subtypes. Given that, particularly PermEn2D, SampEn2D, and FuzzEn2D were selected by SFM for further classification, one may suggest that specific aspects of subsequent entropy features represent some specific properties of ADC and SCC texture, which is important for their discrimination. PermEn2D represents the patterns of length m derived from ordered vectors reflecting pixel intensities [25]. SampEn2D represents the simple patterns of length m [39]. Meanwhile, FuzzEn2D represents the probability that two patterns similar in their corresponding m points will remain similar in the following m + 1 points [29]. However, the direct relations between entropy–based texture features and histological characteristics of NSCLC, as well as the biological rationale for why PermEn2D was selected for high–performing classification, required further research.

Further research is also required to confirm these findings using a bigger sample size and external validation, since this study is limited by the single–center design, small sample size, and lack of external validation. As in previous similar studies [14,20,36], an internal 5–fold cross–validation was used. However, in this study the cohort size (31 patients) was smaller than in all previous studies, which used MRI images collected from 45 patients [36], 61 patients [14], 71 patients [20], 80 patients [18], and 155 patients [47], and the gender – similarly to previous studies [14,18,20,36,47] – was imbalance. Further limitations result from the MRI slice thickness, which may limit sensitivity to fine–grained tumor heterogeneity. In this study, slice thickness was 4 mm – the same as in one previous study [36], higher than in another previous study (2.8 mm) [14], and lower than in another two previous studies (5 mm) [18,20], except for one study in which slice thickness was not provided [47].

In this single–center study on MRI images, a patient–grouped, 5–fold, cross–validated dataset study design was used, thus providing internally validated results. Among previously published MRI–based radiomic studies on NSCLC subtypes differentiation [14,18,20,36,47], similar 5–folds cross–validated was used in three studies [14,20,36], similar but 10–folds cross–validated was used in one study [47], while in another one study no data on cross–validation was provided [18]. Therefore, the classification performance is further discussed between this study and four previous studies [14,20,36,47] conducted on a similar dataset, extracted using FOS and SOS radiomic approaches, and classified using similar validation. Classification of 61 patients with NSCLC, including 40 ADC and 21 SCC, achieved 0.83 accuracy and 0.85 AUC. In this model, the least absolute shrinkage and selection operator (LASSO) was used for feature selection; however, the ML algorithm used for final classification has not been described [14]. Classification of 71 patients with NSCLC, including 46 ADC and 25 SCC, achieved 0.71 accuracy (71% sensitivity) and 0.86 AUC. In this model, the LASSO was used for feature selection, while the LR algorithm was used for image classification [20]. Classification of 45 patients with NSCLC, including 24 ADC and 21 SCC, achieved 0.70 accuracy and 0.85 AUC. In this model, the random forest algorithm was used for feature selection, while the kNN algorithm was used for image classification [36]. Classification of 155 patients with NSCLC, including 75 ADC and 80 SCC, achieved 0.69 accuracy using the kNN algorithm and 0.75 accuracy using SVM; however, AUC has not been reported. This model used the random forest algorithm for feature selection [47]. Given that SFM protocol used in this study is based on the random forest algorithm [41], the similarity of the feature selection methods used in this study and the two previous studies [36,47] can be confirmed. Additionally, three of these four studies used normalize/Gaussian filtering [14,36,47] for image pre–processing, and only one study did not use pre–processing [20].

This study’s protocol, working on normalize filtered images and the FOS/SOS dataset using the kNN algorithm, achieved 0.62 accuracy and 0.71 AUC, less than reported in a similar study design [36]. This may be due to the smaller sample size in this study and other methods of ROI segmentation, despite the same FOS/SOS (GLCM, GLDM, GLRLM, GLSZM, NGTDM [36]) approaches used. By applying the same protocol (normalize filtration/kNN algorithm) to the combined FOS/SOS/entropy dataset (0.64 accuracy; 0.71 AUC) and entropy–based dataset (0.67 accuracy; 0.73 AUC), the higher performance was achieved; however, still less than in the both previous studies [36,47]. This study’s protocol, working on normalize filtered images and the FOS/SOS dataset using the SVM algorithm, achieved 0.67 accuracy and 0.74 AUC, less than reported in a similar study design [47]. This may be due to the smaller sample size and other datasets used (FOS, SOS (GLCM, GLRLM), model–based methods, and transformation methods [47] vs. GLCM, GLDM, GLRLM, GLSZM, NGTDM). By applying the same protocol (normalize filtration/SVM algorithm) to the combined FOS/SOS/entropy dataset (0.67 accuracy; 0.75 AUC) and entropy–based dataset (0.70 accuracy; 0.75 AUC), a higher performance was achieved; however, still less than previously reported [47]. However, the use of the most efficient protocol in these studies (median filtration/combined FOS, SOS, entropy dataset/LR algorithm) allowed to achieve performance (0.75 accuracy; 0.80 AUC) comparable [47] or higher [20,36] than in all previous similar studies, which allows to assume that applying this protocol to other (larger [20,36,47] or based on more texture features [47]) datasets will allow to obtain even better results. Therefore, it may be suggested that the radiological differentiation between the two most significant histological subtypes of NSCLC [2] may be assessed using entropy–based texture features, and that these features may serve as a complementary diagnostic tool for computer–aided diagnosis of NSCLC subtypes. Given that the sensitivity of histological or cytological examination of tumor samples ranges from 0.73 to 0.98 [30], the obtained findings underscore the potential of advanced tools for extracting and analyzing image features [14–20] to improve the evaluation of MRI images and thus non–invasive differentiation of NSCLC subtypes. Particularly since the selection of appropriate ML algorithms, improving the prediction efficiency, minimizing the risk of overfitting, performing more refined sample data processing, and constructing artificial intelligence classification models are suspected to play an important role in the accurate classification and prediction of cancer [14,50,51], including lung ADC and SCC [14].

## 5 Conclusion

Applying entropy–based ML–supported classification of MRI images enables the differentiation of ADC and SCC. However, combining FOS, SOS, and entropy–based texture features achieved the highest classification performance. The LR algorithm achieved the highest efficiency in this dataset when working on texture features extracted from MRI images after median filtration. The proposed protocol presented higher efficiency than protocols that worked only on the statistical texture features or on the same dataset but on unfiltered and normalize filtered MRI images. Therefore, incorporating the entropy–based texture features – as a complementary diagnostic tool – into the conventional statistical radiomic features may improve the computer–aided diagnosis of NSCLC histological subtypes and may be suggested for further research.
